# Efficient Biocatalytic Synthesis of Chiral Intermediate of Pregabalin Using Immobilized* Talaromyces thermophilus* Lipase

**DOI:** 10.1155/2018/6192059

**Published:** 2018-11-01

**Authors:** Xu Ding, Xiao-Ling Tang, Ren-Chao Zheng, Yu-Guo Zheng

**Affiliations:** ^1^Key Laboratory of Bioorganic Synthesis of Zhejiang Province, College of Biotechnology and Bioengineering, Zhejiang University of Technology, Hangzhou 310014, China; ^2^Engineering Research Center of Bioconversion and Biopurification of Ministry of Education, Zhejiang University of Technology, Hangzhou 310014, China

## Abstract

A mutant L206F/P207F/L259F of* Talaromyces thermophilus* lipase (TTL) exhibited high hydrolytic activity towards 2-carboxyethyl-3-cyano-5-methylhexanoic acid ethyl ester (CNDE) for synthesis of (*S*)-2-carboxyethyl-3-cyano-5-methylhexanoic acid (*S*-CCMA), a key chiral intermediate of pregabalin. However, low conversion at high CNDE concentration and unreusability of the free TTL mutant restricted its industrial applications. In this study, the TTL mutant was immobilized onto epoxy resin and its catalytic properties for kinetic resolution of CNDE were investigated. Under the optimized conditions, the immobilized lipase exhibited an increased catalytic efficiency even at a CNDE concentration of 3 M with 49.7% conversion and 95% *ee*_p_. The conversion retained higher than 46.3% even after 10 times repeated use of the immobilized lipase in* n*-heptane-water biphasic system. These results demonstrated great potential of the immobilized TTL mutant for industrial production of the chiral intermediate of pregabalin.

## 1. Introduction

Lipases (E.C. 3.1.1.3) have been widely employed in organic synthesis for hydrolysis, alcoholysis, acidolysis, esterification, and transesterification of carboxylic acid esters in aqueous or nonaqueous media [1–4]. It was estimated that about 40% of all biotransformations reported to date were performed with lipase. Apart from their biological significance, they play an important role in industries of pharmaceuticals, cosmetic, leather, foods, perfumery, and fine chemicals with advantages of mild reaction conditions, high stability, and broad substrate specificity [5–10].

However, the use of lipases in their soluble or aggregated forms suffered from deactivation and leakage of proteins in aqueous systems, restricting their industrial applications. Immobilization techniques were regarded as the most promising methods for versatile lipase applications with the advantages of continuous use, easy separation, and prevention of protein or microbial contamination [11–15]. Compared with free enzymes, immobilized enzymes also exhibit improved stability and recyclability [12, 16–23]. Due to the high chemical stability and excellent mechanical strength, covalent binding interactions between enzyme and epoxy carrier can remarkably enhance the stability of free enzyme compared with physical or ionic binding and are employed as one of the most popular immobilization methods to improve catalytic performance [24]. For instance, the fermentation broth of mutant* Malassezia globose* lipase was directly immobilized onto epoxy resin ECR8285, which showed excellent reusability, stability and activity towards partial glycerides [25].* Rhizopus arrhizus* lipase covalently attached to epoxy resin SEPABEADS® EC-EP exhibited much higher operational stability for synthesis of (*S*)-2-(1-hydroxy-3-butenyl)-5-methylfuran compared to that of free enzyme [26].

Pregabalin is a marketed GABA (*γ*-aminobutyric acid) analogue for the treatment of epilepsy, neuropathic pain, fibromyalgia, and generalized anxiety disorder in adults [27]. Due to increasing demand of pregabalin, a large number of chemical and enzymatic routes have been developed [28, 29]. (*S*)-2-Carboxyethyl-3-cyano-5-methylhexanoic acid (*S*-CCMA) is the key chiral intermediate of pregabalin. Kinetic resolution of 2-carboxyethyl-3-cyano-5-methylhexanoic acid ethyl ester (CNDE) using commercial lipase Lipolase®and Lipozyme TLIM® as biocatalyst seems to be promising approaches to prepare* S*-CCMA [28]. However, both of them exhibited inferior catalytic efficiency towards CNDE and could not be reused, leading to high cost of the bioprocess.

In our previous work, a mutant L206F/P207F/L259F of TTL was generated by rational design of its crevice-like binding site, which exhibited a 37.23-fold improvement in specific activity for CNDE over the wild-type TTL [30]. However, the free TTL mutant showed low conversion at high CNDE concentration and the unrecyclability of free lipase increased production cost. Herein, in order to realize repeated use of biocatalysts for* S*-CCMA production, the triple mutant of TTL was immobilized onto the epoxy resin D5730 for kinetic resolution of CNDE. Biochemical properties of the immobilized TTL mutant were characterized, and enantioselective hydrolysis of CNDE by immobilized lipase was investigated. The results indicated that the immobilized TTL mutant exhibited a specific activity of 413.2 U/g and the conversion of CNDE reached 46.3% at a substrate loading of 1 M after 10 reaction cycles, demonstrating its excellent catalytic performance.

## 2. Materials and Methods

### 2.1. Materials

The epoxy resin ES-103B was purchased from Nankai Hecheng Technology Co., Ltd (Tianjin, China). The epoxy resin D5730, ECR8204M, D5759, and ECR8204F were purchased from the Purolite Co., Ltd (Huzhou, China). The epoxy resin LX-1000EP was purchased from Sunresin Technology Co., Ltd. Racemic CNDE was prepared according to Zheng et al. [31]. All other chemicals were of analytical grade.

### 2.2. Expression and Immobilization of TTL Mutant

The TTL mutant gene was overexpressed in* Pichia pastoris *X33 [30]. Cells were cultivated in YPD medium (2% peptone, 1% yeast extract, 2% glucose, 50 *μ*g mL^−1^ Zeocin™) at 30°C. The cells were transferred into BMGY medium containing 2% peptone, 1% yeast extract, 1% glycerol, 1.34% yeast nitrogen base, 4×10^−5^% biotin, and 100 mM potassium phosphate buffer (pH 6.0). The cultivation was performed at 30°C for 22 h and after then the cells were harvested by centrifugation, resuspended and cultured in BMMY media containing 2% peptone, 1% yeast extract, 1.34% yeast nitrogen base, 4×10^−5^% biotin, and 100 mM potassium phosphate buffer (pH 6.0) at 30°C. Methanol (2%, v/v) was fed to the BMMY media at 24 h interval for 5 days.

The fermentation broth was centrifuged at 8,000 ×* g* for 10 min at 4°C, and the crude enzyme was covalently immobilized onto the epoxy resin. The epoxy resin (1 g) was added to 25 mL of the crude lipase (0.2 mg/mL) under gentle stirring at room temperature. After immobilization, the resins were vacuum filtrated, washed twice by distilled water. Finally, the immobilized lipase was dried at room temperature and stored at 4°C.

### 2.3. Protein Concentration Assay

The concentration of protein was measured by Coomassie brilliant blue method with bovine serum albumin (BSA) as standard [34]. The immobilization yield was determined using equation:

Immobilization yield (%) = (P_b_-P_a_) / P_b_ × 100.

P_b_ is the amount of protein in supernatant before immobilization and P_a_ is the amount of protein in supernatant after immobilization.

### 2.4. Immobilized Lipase Activity Assay

The standard reaction mixture of enzyme activity assay consisted of 10 mL Tris-HCl buffer (100 mM, pH 8.0), 5 mM zinc acetate, 100 mM CNDE, and 0.2 g immobilized lipase. Zinc ions can significantly suppress product inhibition by forming a complex with CCMA that remained suspended in the emulsion and prevented the inactivation of the enzyme in the reactions [35]. The reaction was carried out at 35°C. One unit of immobilized lipase activity was defined as the amount of enzyme required for releasing 1 *μ*mol* S*-CCMA per minute. The enantiomeric excess of substrate (*ee*_s_) and product (*ee*_p_) was measured by gas chromatography (GC) equipped with the FID detector and capillary column Astec CHIRALDEX™ G-TA (30 m × 0.25 mm, 0.25 *μ*m film thickness) using helium as carrier gas [36]. The conversion (*c*) and enantiomeric ratio (*E*) were calculated based on *ee*_s_ and *ee*_p_ [37].

### 2.5. Effect of pH on Lipase Activity and Stability

The effect of pH on activity of immobilized and free TTL mutant was measured using the following buffers at 100 mM: citric acid/sodium citrate buffer (pH 6.0–6.6), Tris-HCl buffer (pH 7.0–8.9), and Gly-NaOH buffer (pH 9.0-10.0).

The pH stability of the lipase was investigated by incubating the immobilized and free TTL mutant in Tris-HCl buffer (100 mM, pH 8.0) for 2 d, 4 d, 6 d, 8 d, 10 d, 15 d, and 20 d at 4°C.

### 2.6. Effect of Temperature on Lipase Activity and Thermostability

The effect of temperature on lipase activity was investigated at temperatures ranging from 25°C to 70°C under the standard conditions. The reaction was performed at the corresponding temperatures in Tris-HCl buffer (100 mM, pH 8.0). The thermostability of immobilized and free TTL mutant was examined by measuring their residual activity after incubation at 35°C and 50°C, respectively. The initial enzyme activity was taken as 100%. The half-life of the enzyme activity was calculated based on the plots of Ln (RA, relative activity) versus time.

### 2.7. Kinetic Resolution of CNDE by Immobilized TTL Mutant

The reaction mixture (30 mL) contained 8% (w/v) immobilized lipase, 50-150 mM zinc acetate, and 1-3 M CNDE. The reaction was carried out at 30°C and stirred at 500 rpm, and the pH value was maintained at 7.0 using 4 M NaOH solution by Metrohm titration. The conversion and* ee* value were determined by GC analysis.

### 2.8. Reusability of Immobilized TTL Mutant for Hydrolysis of CNDE

Enantioselective hydrolysis of CNDE by immobilized TTL mutant was performed in 30 mL reaction mixture (pH 8.0) containing 8% (w/v) immobilized TTL mutant, 50 mM zinc acetate and 1 M CNDE. The reaction was carried out at 30°C and stirred at 500 rpm. The pH of the reaction was maintained at 7.0 using 4 M NaOH solution by Metrohm titration. The immobilized TLL was recovered by vacuum filtration and then washed twice by water. The recovered immobilized enzyme was dried at room temperature for next cycle.

## 3. Results and Discussions

### 3.1. Selection of Epoxy Resins for Immobilization

**Figure 1:**
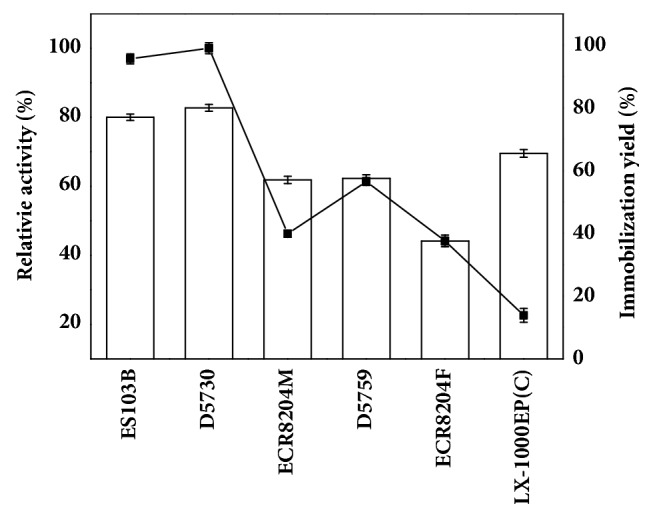
The effect of different resins on immobilization yield and activity of immobilized enzyme. Epoxy resin was directly added to the crude fermentation lipase under gentle stirring at room temperature. The reaction mixture consisted of 10 mL Tris-HCl buffer (100 mM, pH 8.0), 5 mM zinc acetate, 100 mM CNDE, and 0.2 g immobilized lipase. The reaction was performed at 30°C and stirred at 500 rpm. Symbols: filled square, relative activity of immobilized lipase; bar, immobilization yield of epoxy resin.

The effects of epoxy resin carriers on immobilization yield and activity of TTL mutant were shown in Figure 1. When the epoxy resin ES103B and D5730 were used as carriers, more than 75% of lipases in the fermentation broth were immobilized. The TTL mutant immobilized onto epoxy resin D5730 exhibited higher hydrolytic activity than that immobilized onto epoxy resin ES103B. As such, the epoxy resin D5730 with a particle size of 150-300 *µ*m and a pore diameter of 500-600 Å was selected as the carrier for immobilization.

### 3.2. Immobilization of TTL on Epoxy Resin D5730

**Figure 2:**
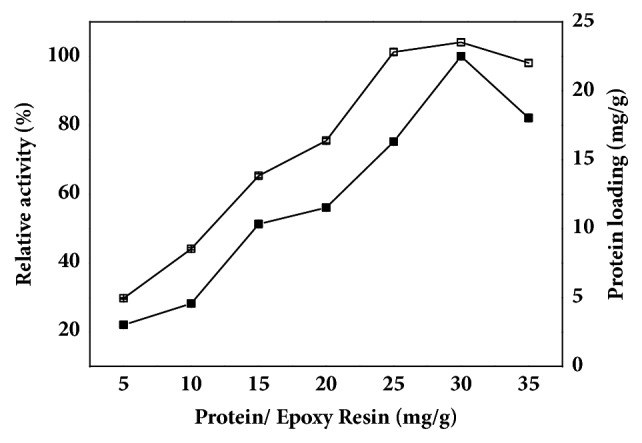
Effect of protein/epoxy resin ratio on immobilization yield and activity of the immobilized TTL mutant. The epoxy resin D5730 was directly added to the crude lipase with different protein/epoxy resin ratio under gentle stirring at room temperature. The reaction was performed at 30°C and stirred at 500 rpm using immobilized enzyme with 100 mM CNDE and 5 mM zinc acetate in Tris-HCl buffer. Symbols: filled square, relative activity of immobilized enzyme; square, protein loading.

In previous works, the immobilization of enzymes onto epoxy supports was preferentially carried out at alkaline pH (around 10) to promote the deprotonation of terminal amino group (pK_a_=6.5) and amino groups of lysine residues (pK_a_=10.5). However, we found that initial pH of the buffer had no significant effect on immobilization efficiency, which was in accordance with immobilization of lipase SMG1 [25]. Moreover, the free TTL mutant was unstable at pH 10.0. Therefore, the crude TTL mutant from fermentation broth was directly immobilized onto the epoxy resin D5730. The effect of protein/epoxy resin ratio on TTL mutant immobilization was investigated. As shown in Figure 2, the maximum loading and highest activity of immobilized lipase was achieved at protein/epoxy resin ratio of 30 mg/g, and the loading of enzyme reached 23.5 mg per gram of epoxy resin. Under this condition, the specific activity of immobilized lipase reached 413.2 U/g carriers with an immobilization yield of 78.3%. Notably, enantioselectivity of the immobilized TTL mutant was as high as that of the free TTL mutant (*E* > 120).

### 3.3. Effect of pH on Lipase Activity and Stability

**Figure 3:**
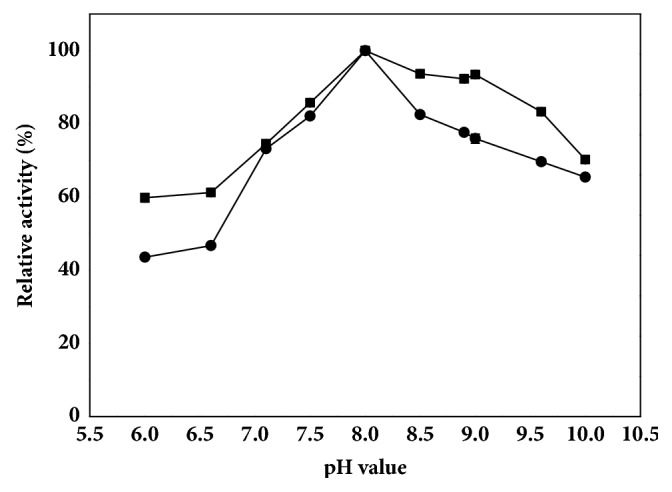
Effect of pH on activity of immobilized and free TTL mutant. The reactions were all carried out at 35°C and the enzyme activities were determined in buffers with different pHs: citric acid/sodium citrate (pH 6.0-6.6), Tris-HCl (pH 7.0-9.0), and Gly-NaOH (9.6-10.0) which contained 100 mM CNDE and 5 mM zinc acetate. Symbols: filled square, immobilized TTL mutant; filled circle, free TTL mutant.

The pH is an important factor in maintaining the proper conformation of a protein [38]. Effects of pH on hydrolytic activity of the free and immobilized TTL mutant were shown in Figure 3. The highest activity of immobilized enzyme was observed around pH 8.0, which was consistent with the free TTL mutant. Moreover, the immobilized TTL mutant displayed higher activity than the free lipase at pH 8.0-9.0, indicating that the immobilized lipase was able to tolerate a broader range of pH.

The pH stability of the free and immobilized TTL mutant was performed in Tris-HCl buffer (100 mM, pH 8.0). Both of them showed excellent stability under the optimal pH 8.0. The immobilized enzyme and free enzyme remained 96% of their initial activity after 20 d incubation at 4°C.

### 3.4. Effect of Temperature on Lipase Activity and Thermostability

**Figure 4:**
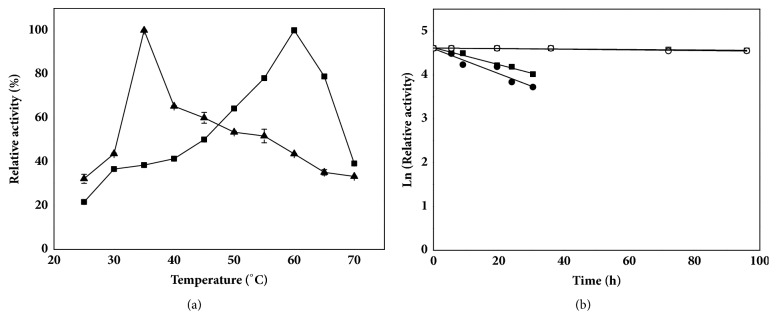
Effect of temperature on activity (a) and thermostability (b) of immobilized and free TTL mutant. The reaction mixture (pH 8.0) was composed of 100 mM Tris-HCl, 100 mM CNDE and 5 mM zinc acetate. Symbols: filled triangle, free TTL mutant; filled diamond, immobilized TTL mutant; filled square, immobilized TTL mutant at 50°C; filled circle, free TTL mutant at 50°C; square, immobilized TTL mutant at 35°C; circle, free TTL mutant at 35°C.

The effect of temperature on activity of immobilized lipase was investigated at a temperature range from 25°C to 70°C. As shown in Figure 4(a), the optimal temperature of the immobilized TTL mutant was 60°C, which was 25°C higher than that of the free TTL mutant. Simultaneously, the immobilized lipase showed broad temperature stability at 55-65°C and retained 75% of its activity at 65°C. The reason for higher activity of the immobilized enzyme over a wider range of temperature may lie in the limited conformational mobility of the enzyme molecules at high temperature, preventing it from inactivation [38].

The thermostability of the immobilized enzyme was investigated by measuring its residual activity after incubation for various periods of time at 35°C and 50°C. As shown in Figure 4(b), after 9 h incubation, the residual activity of the immobilized and free TTL was 89.4% and 68.8% of their initial activity at 50°C, respectively. Both of the immobilized and free TTL mutant remained over 94% of their initial activity after 72 h at 35°C. The half-lives of immobilized and free TTL mutant were determined to be 35.7 h and 24.7 h at 50°C, respectively. The stabilization factor of immobilized TTL mutant ranged from 0.9 to 1.1[39]. The changed optimal temperature and thermostability of immobilized enzyme may attribute to the rigid structure of protein that covalently attached onto the epoxy resin after immobilization [25, 26]. Therefore, the immobilized TTL mutant exhibited higher temperature tolerance for CNDE hydrolysis.

### 3.5. Kinetic Resolution of CNDE by Immobilized Lipase

**Figure 5:**
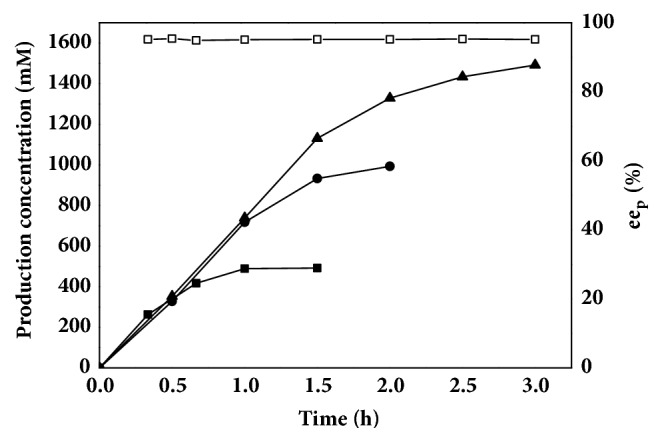
Progress curves of kinetic resolution of CNDE catalyzed by immobilized TTL mutant in Tris-HCl buffer. Symbols: filled square, 1 M CNDE; filled circle, 2 M CNDE; filled triangle, 3 M CNDE; square, *ee*_p_ of* S*-CCMA.

Efficient and enantioselective hydrolysis of CNDE is the most crucial step in chemoenzymatic synthesis of pregabalin [32, 36, 40]. Although the catalytic efficiency of TTL mutant was 89.4 times higher than the commercial lipase Lipolase®, the conversion was low when concentration of CNDE exceeded 1.0 M [30]. The concentration of* S*-CCMA reached 492 mM at a substrate loading of 1 M after 1 h and 993 mM at 2 M CNDE after 2 h, respectively (Figure 5). Notably,* S*-CCMA was produced with 49.7% conversion and > 95%* ee*_*p*_ at a substrate loading up to 3 M after 3 h. Moreover, the reaction time for kinetic resolution of 3 M CNDE by immobilized TTL mutant was shortened from 24 h to 3 h compared with the bioprocess using Liploase® as biocatalysts [28]. The efficient enantioselective hydrolysis of CNDE by the immobilized TTL mutant demonstrated its potential for industrial production of pregabalin precursor (Table 1).

### 3.6. Reusability of the Immobilized Lipase for Hydrolysis of CNDE

**Figure 6:**
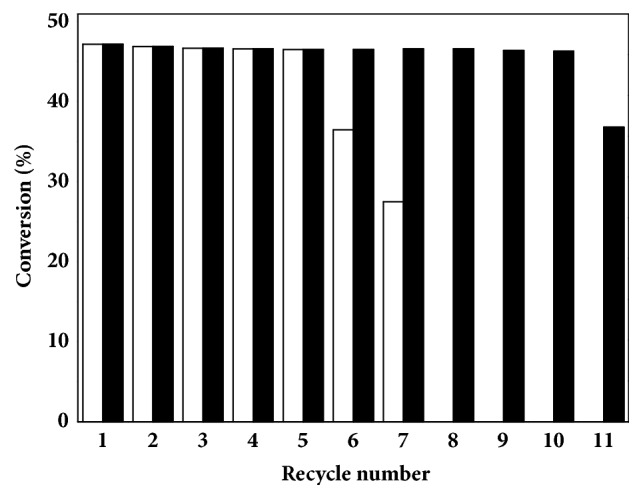
Reusability of immobilized TTL mutant for hydrolysis of CNDE in aqueous and* n*-heptane-water (2:8) biphasic system. The reaction mixture was composed of Tris-HCl buffer or* n*-heptane-water (2:8), 1.0 M CNDE, and 50 mM zinc acetate. The reaction was carried out at 30°C and 500 rpm with mechanical stirring. Symbols: bar, immobilized lipase in aqueous medium; filled bar, immobilized TTL lipase in* n*-heptane-water (2:8) biphasic system.

Except for activity and enantioselectivity, reusability of immobilized enzyme was another important criterion, especially in industrial application [26]. To evaluate the repeated use of the immobilized TTL mutant, the reaction was firstly carried out in aqueous medium with a substrate loading of 1 M. After the reaction was terminated, the immobilized TTL mutant was filtrated under vacuum. As shown in Figure 6, the conversion of CNDE was only 37.5% after 6 times repeated use of immobilized TTL mutant. However, when the biotransformation was performed in* n*-heptane-water (2:8) biphasic system, the conversion was higher than 46.3% after 10 cycles. The results indicated that the immobilized lipase possessed good operational stability in nonaqueous medium.

According to the previous reports, immobilization of enzyme via covalent attachment usually maintained its inherent activity even under harsh conditions [24]. As the substrate CNDE is water insoluble oil, when the reaction was carried out in aqueous system, remaining (*R*)-CNDE was adsorbed on the surface of epoxy resin. With repeated use of the immobilized lipase, more and more unreacted CNDE was accumulated, leading to increased mass transfer resistance and lower catalytic efficiency in the following recycles, while in the* n*-heptane-water biphasic system, unhydrolyzed CNDE was dissolved into the organic phase and the mass transfer problem was thus resolved. However, the conversion of CNDE was only 36.8% after 11 cycles even in biphasic system due to agglomeration of immobilized enzyme caused by viscous unreacted substrate.

## 4. Conclusion

In our study, a mutant L206F/P207F/L259F of* T. thermophilus* lipase was efficiently immobilized onto epoxy resin D5730 and the catalytic properties of the immobilized lipase were characterized. It exhibited excellent catalytic efficiency even at high CNDE concentration of 3 M and showed good reusability in* n*-heptane-water biphasic system. These findings suggested that the immobilized lipase was a robust biocatalyst for industrial production of chiral intermediate of pregabalin.

## Figures and Tables

**Table 1:** Comparison of CNDE resolution between immobilized TTL mutant and other lipases.

Enzyme source	Substrate concentration (M)	Conversion	Space-time yield	Reference
		(%)	(g/L/h)	
*Thermomyces lanuginosus *lipase	3	47.5	12.9	[28]
*Thermomyces lanuginosus* lipase	3	45.2	12.8	[32]
*Pseudomonas* esterase	0.5	47.7	7.7	[33]
*Talaromyces thermophilus *lipase	3	49.7	112.8	This work

## Data Availability

The data used to support the findings of this study are available from the corresponding author upon request.
